# Comparative effectiveness of BNT162b2 and ChAdOx1 nCoV-19 vaccines against COVID-19

**DOI:** 10.1186/s12916-023-02795-w

**Published:** 2023-02-28

**Authors:** Jie Wei, Weiya Zhang, Michael Doherty, Zachary S. Wallace, Jeffrey A. Sparks, Na Lu, Xiaoxiao Li, Chao Zeng, Guanghua Lei, Yuqing Zhang

**Affiliations:** 1 Health Management Center, Xiangya Hospital, Central South University, Changsha, China; 2 Academic Rheumatology, Clinical Sciences Building, University of Nottingham, City Hospital, Nottingham, UK; 3 Arthritis Research UK Pain Centre, Nottingham, UK; 4 Division of Rheumatology, Allergy, and Immunology, Department of Medicine, Massachusetts General Hospital, Harvard Medical School, Boston, USA; 5 The Mongan Institute, Massachusetts General Hospital, Harvard Medical School, Boston, USA; 6 Department of Medicine, Harvard Medical School, Boston, USA; 7 Division of Rheumatology, Inflammation, and Immunity, Brigham and Women’s Hospital, Boston, USA; 8 Arthritis Research Canada, Richmond, Canada; 9 National Clinical Research Center for Geriatric Disorders, Xiangya Hospital, Central South University, Changsha, China; 10 Department of Orthopaedics, Xiangya Hospital, Central South University, Changsha, China

**Keywords:** COVID-19, Vaccine, BNT162b2, ChAdOx1 nCoV-19

## Abstract

**Background:**

Both BNT162b2 (Pfizer–BioNTech) and ChAdOx1 nCoV-19 (Oxford–AstraZeneca) vaccines have shown high efficacy against COVID-19 in randomized controlled trials. However, their comparative effectiveness against COVID-19 is unclear in the real world. We evaluated the comparative effectiveness of the BNT162b2 and ChAdOx1 nCoV-19 vaccines against COVID-19 in the UK general population.

**Methods:**

We emulated a target trial using IQVIA Medical Research Database (IMRD), an electronic primary care database from the UK (2021). We included 1,311,075 participants, consisting of 637,549 men and 673,526 women age≥18 years, who received vaccination with BNT162b2 or ChAdOx1 nCoV-19 between January 1 and August 31, 2021. The outcomes consisted of confirmed diagnosis of SARS-CoV-2 infection, hospitalisation for COVID-19 and death from COVID-19 in the IMRD. We performed a cox-proportional hazard model to compare the risk of each outcome variable between the two vaccines adjusting for potential confounders with time-stratified overlap weighting of propensity score (PS).

**Results:**

During a mean of 6.7 months of follow-up, 20,070 confirmed SARS-CoV-2 infection occurred in individuals who received BNT162b2 vaccine (PS weighted incidence rate: 3.65 per 1000 person-months), and 31,611 SARS-CoV-2 infection occurred in those who received ChAdOx1 nCoV-19 vaccine (PS weighted incidence rate: 5.25 per 1000 person-months). The time-stratified PS weighted rate difference of SARS-CoV-2 infection for BNT162b2 group vs. ChAdOx1 nCoV-19 group was -1.60 per 1000 person-months (95% confidence interval [CI]: -1.76 to -1.43 per 1000 person-months), and the hazard ratio was 0.69 (95% CI: 0.68 to 0.71). The results were similar across the stratum of sex, age (<65 and ≥65 years), and study periods (i.e., alpha-variant predominance period and delta-variant predominance period). The PS weighted incidence of hospitalisation for COVID-19 was also lower in the BNT162b2 vaccine group than that in the ChAdOx1 vaccine group (RD: -0.09, 95%CI: -0.13 to -0.05 per 1000 person-months; HR: 0.65, 95%CI: 0.57 to 0.74). No significant difference in the risk of death from COVID-19 was observed between the two comparison groups.

**Conclusions:**

In this population-based study, the BNT162b2 vaccine appears to be more efficacious than the ChAdOx1 nCoV-19 vaccine against SARS-CoV-2 infection and hospitalisation for COVID-19 but not death from COVID-19.

**Supplementary Information:**

The online version contains supplementary material available at 10.1186/s12916-023-02795-w.

## Background

Covid-19 vaccination is critical for controlling the COVID-19 pandemic. The United Kingdom (UK) implemented a COVID-19 vaccination program after the emergency use approval of the Pfizer-BioNTech messenger RNA (mRNA) vaccine, BNT162b2, in December 2020 [1]. Later, the vaccination program was expanded to include the Oxford-AstraZeneca adenovirus (AdV) vector vaccine, ChAdOx1 nCoV-19 [2] (hereafter referred to ChAdOx1), and Moderna mRNA vaccine mRNA-1273 [3]. To date, the majority have received two doses of either BNT162b2 or ChAdOx1 vaccines in the UK [4, 5]. To date, the percentage of fully vaccinated population remains low (<20%) in most countries of Africa and several countries of West Asia [6].

Previous studies reported that the AdV vector vaccine might induce higher levels of specific T cells, whereas the mRNA vaccine might induce higher antibody titers [7–9]. Results from the phase III clinical trials showed 95% efficacy for BNT162b2 [10] and 70% efficacy for ChAdOx1 [11] against COVID-19 after two vaccine doses. However, the results from the observational studies were inconsistent when the effectiveness of these two vaccines was compared with non-vaccination. Some cohort studies [12–15] and a test negative case-control study [16] showed higher effectiveness against COVID-19 for the BNT162b2 vaccine than the ChAdOx1 vaccine when compared with unvaccinated individuals, whereas other cohort studies [4, 17–19] and test negative case-control study [20] found no apparent difference in the effectiveness of these two vaccines against COVID-19 compared with unvaccinated individuals. To date, there is a paucity of evidence of head-to-head comparisons of these two vaccines, leaving knowledge gaps regarding which vaccine is more effective against COVID-19.

Therefore, we emulated a target trial to evaluate the comparative effectiveness of the BNT162b2 vaccine vs. the ChAdOx1 vaccine against COVID-19 using data from a UK primary care database.

## Methods

### Data sources

We used data from the IQVIA Medical Research Database (IMRD) (previously called The Health Improvement Network (THIN)), an electronic health records database of general practitioner (GP) based healthcare covering 839 practices and 19 million individuals in the UK. The IMRD was established in 2003 as a collaboration between the company owning Vision (In Practice Systems) and the CSD Medical Research Group (now Quintiles IMS). The database contains computerised information on socio-demographics, anthropometric characteristics, lifestyle factors, and details from visits to GPs (i.e., prescriptions, diagnosis, diagnoses and interventions from specialist referrals, hospital admissions, and results of laboratory tests). The READ classification system is used to code specific diagnoses [21], whereas a dictionary based on the Multilex classification system is used to code drugs [22]. The validity of the IMRD for use in clinical and epidemiological research studies has been demonstrated in a previous study [23]. The scientific review committee for the IMRD database and the institutional review board at Xiangya Hospital approved this study, with a waiver of informed consent. This study followed the recommendations of the STROBE initiative for reporting observational studies in epidemiology [24].

### Study design and cohort definition

We emulated a target trial to compare the risk of SARS-CoV-2 infection and its sequelae, i.e., hospitalisation and death in individuals who received the BNT162b2 vaccine with those who received the ChAdOx1 vaccine. We included individuals between 18 to 89 years of age, who received their first covid vaccination with either of these two vaccines from January 1, 2021, to August 31, 2021, and had at least two years of continuous enrolment with a general practice prior to entering the study. The details of vaccination records were based on the READ code in IMRD (Additional file 1: Table S1) [22]. The date of the first dose of either the BNT162b2 vaccine or the ChAdOx1 vaccine was assigned as the index date. Individuals were excluded if they had received a SARS-CoV-2 infection diagnosis or a different COVID-19 vaccine (i.e., mRNA-1273 [Moderna] and Ad26.CoV2.S [Janssen/Johnson &Johnson]) prior to the index date.

### Emulation of the target trial

**Fig. 1:**
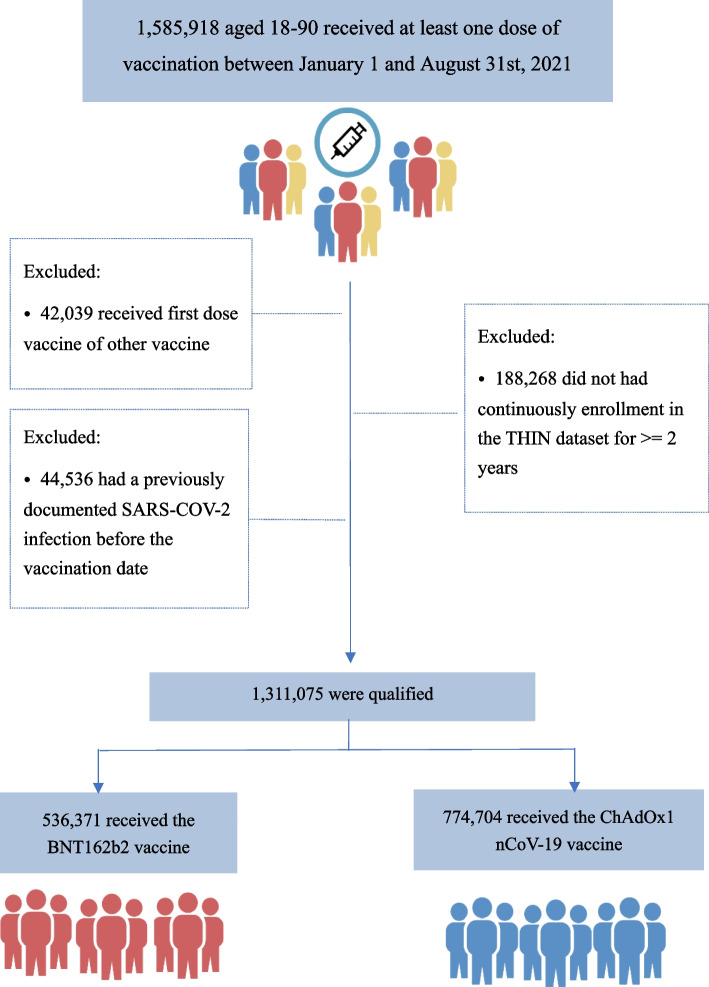
Selection process of participants for the emulation of a target trial evaluating the comparative effectiveness of the BNT162b2 and ChAdOx1 nCoV-19 vaccines

We divided the baseline study period into weekly time blocks. Eligible individuals were allocated into these blocks according to their index dates. In each weekly time block we calculated the propensity score (PS) and adopted an overlap weighting approach to balance baseline characteristics (Fig. 1) [25, 26]. Specifically, the PS for the BNT162b2 vaccine was calculated in each weekly time block using a logistic regression model that included potential confounders. Individuals receiving the BNT162b2 vaccine were weighted by the probability of not receiving the BNT162b2 vaccine, i.e., 1-PS, and individuals receiving the ChAdOx1 vaccine were weighted by the probability of receiving the BNT162b2 vaccine, i.e., PS. Overlap weights were bounded and smoothly reduced the influence of individuals at the tails of the PS distribution without making any exclusions.

### Assessment of outcome

The primary outcome was a confirmed diagnosis of SARS-CoV-2 infection, and the secondary outcomes were hospitalisation for COVID-19 and death from COVID-19. A confirmed diagnosis of SARS-CoV-2 infection was based on READ codes recommended in national guidelines (Additional file 1: Table S1) [27, 28]. According to National Health Service Guidance and Standard Operating Procedures for Primary Care, and the UK Faculty of Clinical Informatics guidelines, confirmed SARS-CoV-2 infection codes represent a positive RT-PCR test [29]. Hospitalisation for COVID-19 was defined as a hospitalisation record in the IMRD [30] within 30 days after documentation of SARS-CoV-2 infection, and death from COVID-19 was defined as a death within 30 days after documentation of SARS-CoV-2 infection.

### Assessment of negative control outcome

We used two negative control outcomes (i.e., SARS-CoV-2 infection occurred over the 14 days after receiving the first dose of the COVID-19 vaccine and death from cardiovascular disease [CVD] defined as a death within 30 days before or after documentation of myocardial infarction, stroke or heart failure) to evaluate for potential residual confounding [31]. No difference in the risk of the outcomes between two comparison groups should be observed unless there were residual confounders (e.g., health status or healthcare-seeking behaviour).

### Assessment covariates

The variables in the PS model consisted of sociodemographic factors (i.e., age, sex, Townsend Deprivation Index), geographic location, race, body mass index (BMI), lifestyle factors (i.e., alcohol use and smoking status), influenza vaccination history during the past one year before the index date, comorbidities prior to the index date (i.e., hypertension, diabetes, chronic kidney disease, pneumonia or infection, chronic obstructive pulmonary disease, influenza, cancer, venous thrombosis, atrial fibrillation, ischemic heart disease, congestive heart failure, stroke, trauma, fracture, liver disease, fall, dementia, and depression), medication use (antidiabetic, antihypertensive, statin, diuretics, glucocorticoids, non-steroidal anti-inflammatory drugs, opioids, proton-pump inhibitors, biologic disease modifying antirheumatic drugs) and healthcare utilization during the past one year immediately before the index date. A directed acyclic graph of the comparative effectiveness of BNT162b2 and ChAdOx1 nCoV-19 vaccines against COVID-19 and potential confounders was shown in Additional file 1: Figure S1.

### Statistical analysis

The baseline characteristics of individuals who received the BNT162b2 vaccine were compared with individuals who received the ChAdOx1 vaccine using standardized difference. For the primary outcome, person-months of follow-up for each individual were calculated as the amount of time from the index date to the first of the following events: confirmed diagnoses of SARS-CoV-2 infection, death, disenrollment from a GP practice participating in IMRD, 10 months follow-up, receiving a different COVID-19 vaccine of the second dose, or the end of the study (October 31, 2021). We calculated the rate of SARS-CoV-2 infection and plotted cumulative incidence curves of SARS-CoV-2 infection for individuals who received the BNT162b2 vaccine and individuals who received the ChAdOx1 vaccine, respectively. We estimated the absolute rate difference (RD) of SARS-CoV-2 infection between the two comparison groups. We obtained the hazard ratio (HR) of SARS-CoV-2 infection for the individuals who received the BNT162b2 vaccine vs. those who received the ChAdOx1 vaccine using Cox proportional hazard model accounting for competing event (i.e., death). We tested the proportional hazard assumption by plotting the cumulative incidence curve of each outcome. We then conducted a weighted cox regression to obtain a non-proportional HR if the proportional hazard assumption was violated [32]. We repeated the analysis to evaluate the comparative effectiveness of the two vaccines for secondary outcomes.

We performed four sensitivity analyses to assess the robustness of the study findings. First, we conducted subgroup analyses to assess the comparative effectiveness of these two vaccines according to sex (male vs. female), age (≥65 vs. <65 years), and study period (i.e., SARS-CoV-2 alpha-variant predominance period from January 1 to May 16, 2021 vs. delta-variant predominance period from May 17 to October 31, 2021 [13]). Second, to evaluate the potential residual confounding effect, we examined the relation of the BNT162b2 vaccine vs. the ChAdOx1 vaccine to the risk of SARS-CoV-2 infection in the first 14 days after the first dose of vaccination and death from CVD, respectively. Third, we performed the analysis by including the participants who had SARS-COV-2 infection prior to the index date. In this analysis, we added the history of previous SARS-COV-2 infection when calculating the PS. Fourth, we assessed the risk of SARS-CoV-2 infection among participants who received two doses of vaccines and one dose of vaccine separately.

All *P* values were 2-sided and P<0.05 was considered significant for all tests. All statistical analyses were performed with SAS software, version 9.4 (SAS Institute, Cary, North Carolina, USA) and R Studio, version 1.1,456 (R Foundation, Vienna, Austria).

## Results

Among 1,585,918 individuals aged 18-90 years who received at least one dose of vaccination between January 1 and August 31, 2021, in the IMRD, 536,371 individuals received the BNT162b2 vaccine and 774,704 received the ChAdOx1 nCoV-19 vaccine. The baseline characteristics of the study population are shown in Table 1. More than 90% of individuals in each group received a second dose of the same vaccine. Before PS overlap weighting, individuals who received the BNT162b2 vaccine were younger, had a lower BMI, a lower percentage of influenza vaccination, comorbidities, and medication use. After PS overlap weighting, all measured baseline characteristics were well-balanced between the two comparison groups (all standardized difference < 0.02).

**Table 1:** Baseline characteristics of individuals newly received the BNT162b2 and the ChAdOx1 vaccine

	Before propensity-score overlap weighting			After propensity-score overlap weighting		
	BNT162b2	ChAdOx1	Standardized difference	BNT162b2	ChAdOx1	Standardized difference
**Number**	536,371	774,704		163,716	163,716	
**Demographics**						
Age, mean (SD), y	46.37 (19.13)	57.82 (14.82)	0.669	58.32 (15.77)	58.32 (16.75)	<0.001
Race (%)			0.075			<0.001
White	42.7	46		45	45	
Other	3.4	2.6		2.8	2.8	
Missing	53.9	51.3		52.2	52.2	
Socioeconomic deprivation index score (%)^a^			0.052			<0.001
1	15.5	15.8		16.7	16.7	
2	18.5	19.7		19.8	19.8	
3	19.7	20.1		19.8	19.8	
4	18.6	18.6		17.7	17.7	
5	15.5	14.9		14.2	14.2	
Missing	12.2	10.9		11.9	11.9	
Female (%)	52.9	50.3	0.052	52.2	52.2	<0.001
**BMI, mean (SD), kg/m**^**2**^	27.44 (5.87)	28.45 (6.30)	0.165	27.98 (5.80)	28.07 (6.00)	0.015
**Region**			0.105			<0.001
England	14.8	13.6		15.1	15.1	
Northern Ireland	15.3	12.3		17.6	17.6	
Scotland	39.1	42.8		38.1	38.1	
Wales	30.8	31.4		29.2	29.2	
**Influenza vaccination (%)**^b^	31.8	45	0.272	49.1	49.1	<0.001
**Lifestyle factors**						
Drinking (%)			0.037			<0.001
None	19.1	18.1		18.2	18.2	
Past	2.9	3.4		3.5	3.5	
Current	77.9	78.5		78.3	78.3	
Smoking (%)			0.134			<0.001
None	62	55.8		56.9	56.9	
Past	21.7	26.8		27.5	27.5	
Current	16.2	17.4		15.6	15.6	
**Comorbidity (%)**						
Hypertension	18.2	28.7	0.249	30.5	30.5	<0.001
Diabetes	8.9	14	0.162	14.1	14.1	<0.001
Chronic kidney disease	3.0	5.0	0.102	5.5	5.5	<0.001
Pneumonia or infection	5.4	6.8	0.060	6.7	6.7	<0.001
Chronic obstructive pulmonary disease	2.3	4.4	0.112	4.6	4.6	<0.001
Influenza	3.1	3.6	0.030	3.6	3.6	<0.001
Cancer	6.3	9.2	0.106	10.4	10.4	<0.001
Venous thrombosis	1.5	2.6	0.079	2.5	2.5	<0.001
Atrial fibrillation	2.2	3.8	0.095	4.1	4.1	<0.001
Ischemic heart disease	4	6.7	0.120	7.3	7.3	<0.001
Myocardial infarction	1.8	3.2	0.088	3.3	3.3	<0.001
Congestive heart failure	1.1	2.0	0.075	2.0	2.0	<0.001
Stroke	1.4	2.6	0.085	2.6	2.6	<0.001
Trauma	0.9	1.1	0.025	1.0	1.0	<0.001
Fracture	31.2	33.3	0.044	32.6	32.6	<0.001
Liver disease	2.2	3.4	0.075	3.4	3.4	<0.001
Fall	5.8	7.8	0.079	8.0	8.0	<0.001
Dementia	0.6	1.1	0.053	1.1	1.1	<0.001
Depression	12.4	15.2	0.083	14.4	14.4	<0.001
**Medication (%)**^b^						
Antihypertensive	22.1	33	0.247	34.8	34.8	<0.001
Antidiabetic	4.5	7.9	0.142	7.6	7.6	<0.001
Statin	15	23.2	0.212	25.9	25.9	<0.001
Loop diuretics	2.0	3.9	0.117	3.8	3.8	<0.001
Thiazide diuretics	3.6	5.5	0.093	6.2	6.2	<0.001
Glucocorticoids	3.3	5	0.084	5.2	5.2	<0.001
NSAIDs	16.1	21.4	0.136	21.2	21.2	<0.001
Opioids	5.0	8.1	0.127	7.6	7.6	<0.001
PPIs	20.1	27.9	0.182	28.5	28.5	<0.001
DMARDs	1.1	1.7	0.051	1.9	1.9	<0.001
**Healthcare utilization, mean (SD)**^b^						
Hospitalizations^b^	0.19 (0.70)	0.24 (0.86)	0.057	0.24 (0.82)	0.24 (0.80)	<0.001
General practice visits^b^	1.69 (3.13)	2.13 (3.95)	0.123	2.13 (3.87)	2.13 (3.53)	<0.001
Specialist referrals^b^	0.21 (0.62)	0.23 (0.66)	0.026	0.24 (0.67)	0.24 (0.69)	<0.001

*BMI* Body mass index, *n* number, *y* years, *SD* Standard deviation, *NSAIDs* Non-steroidal anti-inflammatory drugs, *PPIs* Proton-pump inhibitors, *DMARDs* Biologic disease modifying antirheumatic drugs

^a^The Socio-Economic Deprivation Index was measured by the Townsend Deprivation Index, which was grouped into quintiles from 1 (least deprived) to 5 (most deprived)

^b^Frequency during the past year

**Fig. 2:**
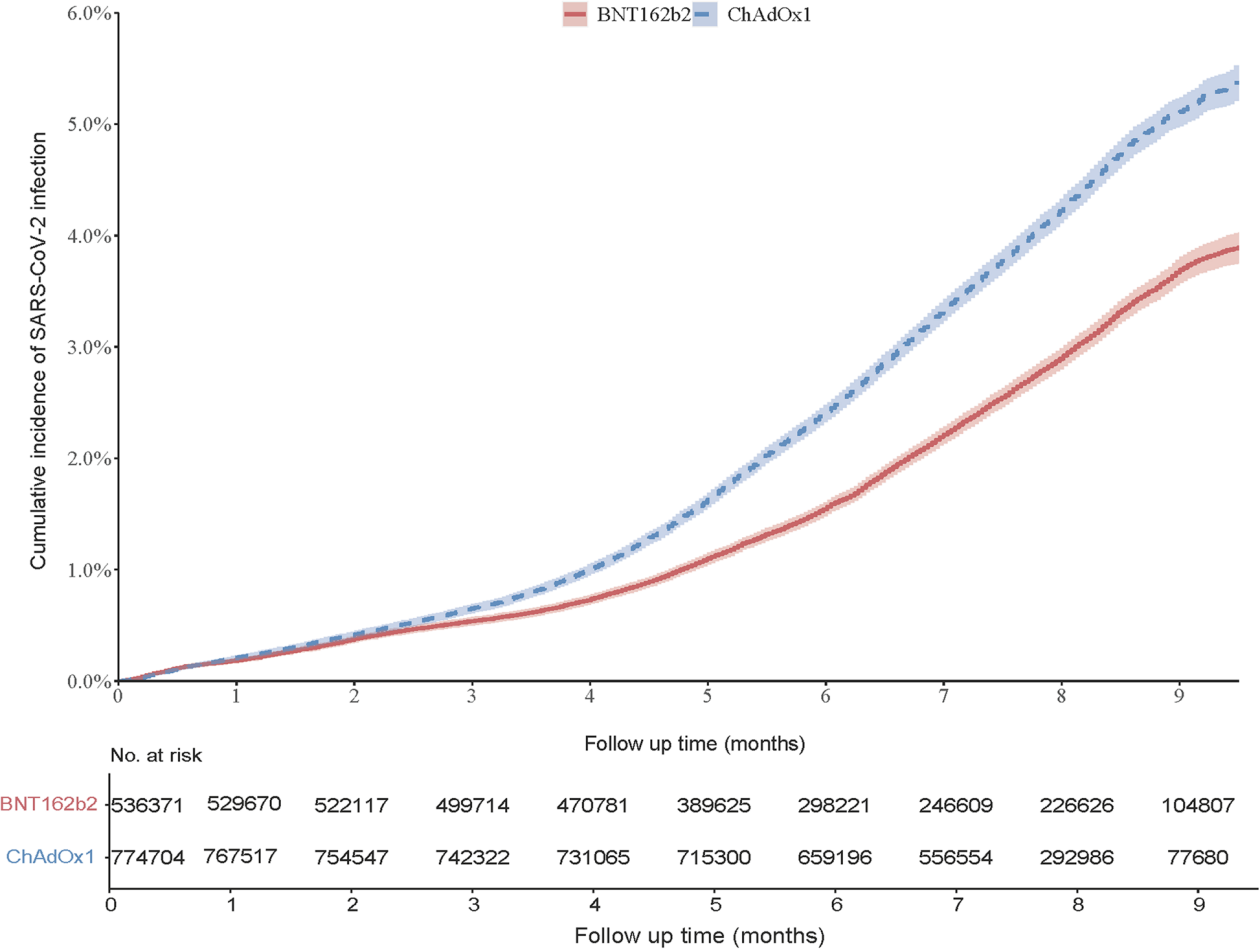
Cumulative incidence of SARS-CoV-2 infection between participants received BNT162b2 and ChAdOx1 nCoV-19 vaccines

During a mean of 6.7 months of follow-up, 20,070 confirmed SARS-CoV-2 infection cases occurred in the BNT162b2 vaccine group, and 31,611 cases occurred in the ChAdOx1 nCoV-19 vaccine group. The weighted incidence of COVID-19 was lower in the BNT162b2 vaccine group (3.65 per 1000 person-months) than that in the ChAdOx1 vaccine group (5.25 per 1000 person-months) (Fig. 2 and Table 2). Compared with those who received the ChAdOx1 vaccine, the RD of SARS-CoV-2 infection among individuals who received the BNT162b2 vaccine was -1.60 (95% confidence interval [CI]: -1.76 to -1.43) per 1000 person-months, and HR was 0.69 (95%CI: 0.68 to 0.71). The results were consistent across the stratum of sex, age (<65 and ≥65 years), and calendar periods (alpha-variant predominance period and delta-variant predominance period), including the individuals who were infected prior to the index date, as well as among participants who received two doses of vaccines. There was no significant difference between BNT162b2 and ChAdOx1 vaccines for SARS-CoV-2 infection among the participants who received only one dose of the vaccine under investigation.

**Table 2:** Estimated comparative effectiveness of the BNT162b2 and ChAdOx1 vaccines for SARS-CoV-2 infection

Population	Number		No. of Events		Mean follow up, months		Incidence rate^a^, per 1000 person-months		Rate difference^a^ (95%CI), per 1000 person-months	Hazard ratio^a^ (95%CI)
	BNT162b2	ChAdOx1	BNT162b2	ChAdOx1	BNT162b2	ChAdOx1	BNT162b2	ChAdOx1		
**Overall**	536,371	774,704	20,070	31,611	6.65	7.33	3.65	5.25	-1.60 (-1.76, -1.43)	0.69 (0.68, 0.71)
**Sex**										
Male	252,646	384,903	8,582	14,666	6.34	7.25	3.42	4.85	-1.44 (-1.67, -1.20)	0.70 (0.68, 0.73)
Female	283,725	389,801	11,488	16,945	6.93	7.40	3.85	5.59	-1.74 (-1.98, -1.50)	0.68 (0.66, 0.71)
**Age**										
Age>=65	132,624	235,594	2,482	4,886	8.49	8.36	2.17	2.72	-0.55 (-0.74, -0.36)	0.79 (0.75, 0.84)
Age<65	403,747	539,110	17,588	26,725	6.07	6.89	5.15	7.62	-2.47 (-2.76, -2.19)	0.67 (0.65, 0.70)
**Study period**										
Alpha-variant predominance	356,679	739,203	1,252	1,227	2.61	2.24	0.84	0.94	-0.10 (-0.23, 0.04)	0.89 (0.80, 1.00)
Delta-variant predominance	179,692	35,501	8,413	1,996	4.31	4.86	8.02	11.99	-3.96 (-5.02, -2.90)	0.67 (0.62, 0.72)
**Including prior infected population**	561,140	803,200	20,398	32,007	6.63	7.32	3.65	5.28	-1.62 (-1.79, -1.47)	0.69 (0.67, 0.71)
**Received two doses**	491,591	730,363	12,229	28,196	6.96	7.58	3.01	4.59	-1.58 (-1.73, -1.42)	0.65 (0.64, 0.67)
**Received one dose**	29,701	21,870	2,138	905	4.26	5.13	9.07	8.91	0.16 (-1.31, 1.64)	1.01 (0.91, 1.14)

*No* Number, *CI* Confidence interval

^a^Estimates were time-stratified overlap weighted of propensity score

**Fig. 3:**
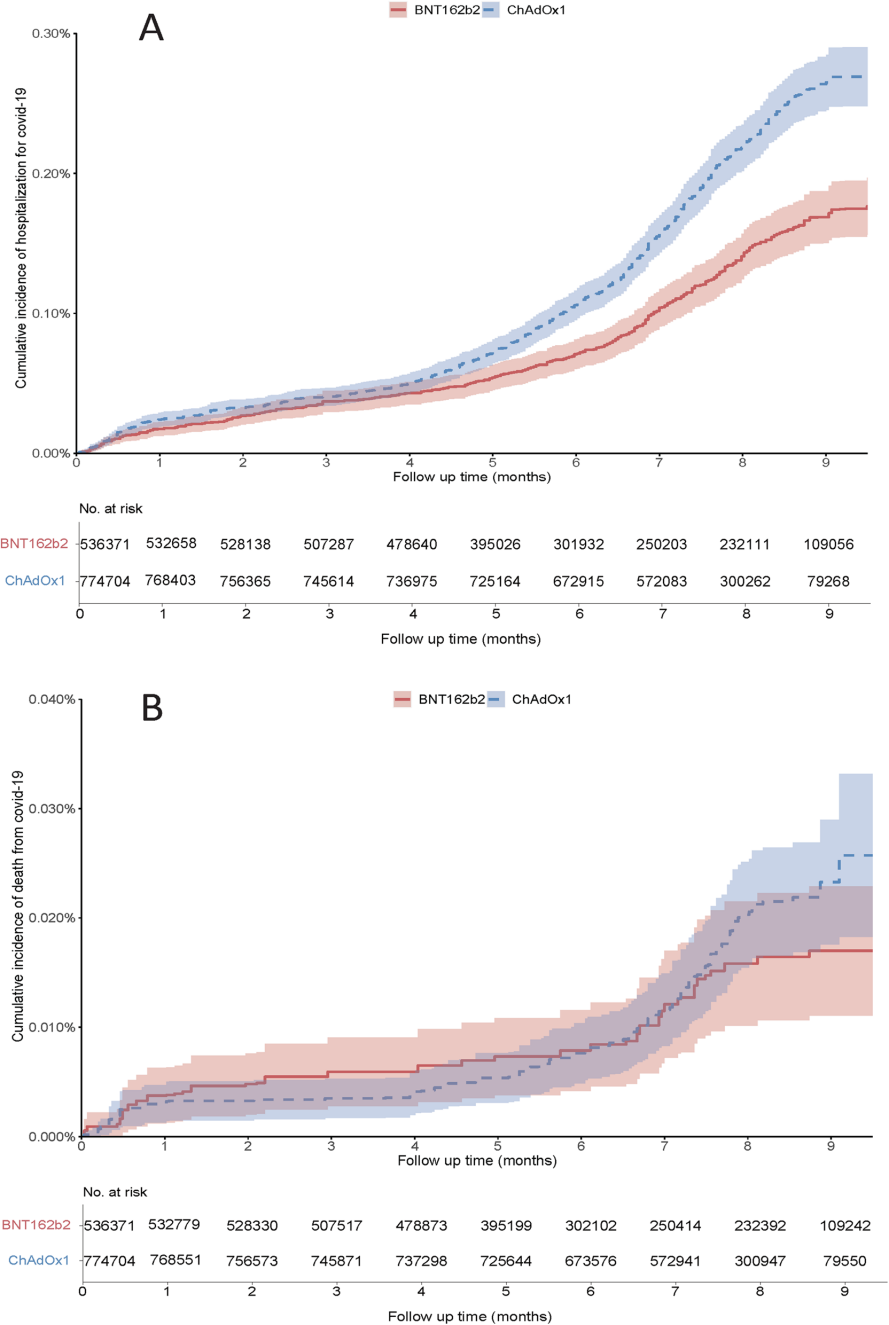
Cumulative incidence of hospitalisation for COVID-19 (A) and death from COVID-19 (B) between participants received BNT162b2 and ChAdOx1 nCoV-19 vaccines

There were 603 and 1,432 hospitalisations for COVID-19 in individuals who received the BNT162b2 vaccine and in those who received the ChAdOx1 nCoV-19 vaccine, respectively. The weighted incidence of hospitalisation for COVID-19 was lower in the BNT162b2 vaccine group (0.17 per 1000 person-months) than that in the ChAdOx1 vaccine group (0.26 per 1000 person-months), and the corresponding RD and HR were -0.09 (95%CI: -0.13 to -0.05) per 1000 person-months and 0.65 (95%CI: 0.57 to 0.74), respectively. The mortality rate from COVID-19 was slightly lower, albeit non-statistically significant, in the BNT162b2 vaccine group (0.018 per 1000 person-months) than in the ChAdOx1 vaccine group (0.022 per 1000 person-months), with corresponding RD and HR being -0.005 (95%CI: -0.016 to 0.006) and 0.66 (95%CI: 0.42 to 1.04), respectively (Fig. 3 and Table 3).

**Table 3:** Comparison of the BNT162b2 and the ChAdOx1 vaccines for hospitalisation for COVID-19 and death from COVID-19

Outcomes	BNT162b2	ChAdOx1
**Number**	536,371	774,704
**Hospitalisation for COVID-19**		
Event (n)	603	1,432
Mean follow-up (months)	6.74	7.41
Incidence rate^a^, per 1000 person-months	0.17	0.26
Rate difference (95%CI) ^a^, per 1000 person-months	-0.09 (-0.13, -0.05)	0.00 (ref)
Hazard ratio (95% CI) ^a^	0.65 (0.57, 0.74)	1.00 (ref)
**Death from COVID-19**		
Event (n)	42	112
Mean follow-up (months)	6.74	7.41
Incidence rate^a^, per 1000 person-months	0.018	0.022
Rate difference (95%CI) ^a^, per 1000 person-months	-0.005 (-0.016, 0.006)	0.00 (ref)
Hazard ratio (95% CI) ^a^	0.66 (0.42, 1.04)	1.00 (ref)

*CI* Confidence interval

^a^Estimates were time-stratified overlap weighted of propensity score

No difference in the risk of SARS-CoV-2 infection during the first 14 days after receiving the first vaccine dose (i.e., negative control outcome) of the BNT162b2 vaccine vs. the ChAdOx1 vaccine was observed. Compared with those who received the ChAdOx1 vaccine, the RD of SARS-CoV-2 infection during the first 14 days after receiving the first vaccine dose among individuals who received the BNT162b2 was 0.20 (95% CI: -0.26 to 0.66) per 1000 person-months, and HR was 1.11 (95% CI: 0.94 to 1.30). Similar null association was observed in the risk of death from CVD after receiving the BNT162b2 vaccine vs. the ChAdOx1 vaccine (HR 0.90, 95%CI: 0.57 to 1.41).

## Discussion

Our study provides timely real-world evidence of the comparative effectiveness of the BNT162b2 and the ChAdOx1 vaccines against the risk of SARS-CoV-2 infection. Individuals who received the BNT162b2 vaccine had a 30% lower risk of SARS-CoV-2 infection than those who received the ChAdOx1 vaccine over 10 months of follow-up. Our findings are consistent across sex, age, and study periods and unlikely to be explained by major residual confounders. In addition, the BNT162b2 vaccine appears to provide a better protective effect on hospitalisation for COVID-19 than the ChAdOx1 vaccine.

### Comparison with previous studies

To date, results from their respective phase III clinical trials demonstrated that the BNT162b2 vaccine had a 95% efficacy in protecting against COVID-19, while the ChAdOx1 vaccine had a 70% efficacy against COVID-19. Consistent with the findings of clinical trials, results from several observational studies also indirectly showed a slightly higher effectiveness of the BNT162b2 vaccine than the ChAdOx1 vaccine when compared with unvaccinated individuals [12–16]. Meanwhile, other observational studies suggested similar effectiveness of the BNT162b2 and ChAdOx1 vaccines against SARS-CoV-2 infection, symptoms, hospital admissions, and death from SARS-CoV-2 infection when compared with unvaccinated individuals [4, 17–20] Our study demonstrated greater effectiveness of the BNT162b2 vaccine, indicated by both absolute (i.e., RD) and relative (i.e., HR) effects on the risk of SARS-CoV-2 infection and hospitalisation for COVID-19 than the ChAdOx1 vaccine over ten months follow-up. Although the infection rate was different between the SARS-CoV-2 alpha-variant predominance period and the delta-variant predominance period, which may be due to a differential vaccine waning, the comparative effectiveness of the BNT162b2 and ChAdOx1 vaccines against SARS-CoV-2 infection was consistent across these two study periods. This real-world empirical data provided critical evidence of the relative effectiveness of these two vaccines in mitigating the COVID-19 burden in the general population [33].

### Potential explanations

Both the mRNA and AdV vector vaccines encode the production of the SARS-CoV-2 spike (S) protein, which is the major target for neutralizing antibodies generated from natural infection and for therapeutic monoclonal antibodies [34–36]. However, the two mechanisms leverage different aspects of the immune response, particularly the innate immune response, which may lead to differences in immunogenicity [34]. Previous studies have reported that the BNT162b2 vaccine might induce higher antibody titres than the ChAdOx1 vaccine [7–9], which may partially explain the differences in the effectiveness against COVID-19 between these two vaccines.

### Strengths and limitations

There are several strengths to our study. First, we emulated a hypothetic trial using a population-based electronic database to evaluate the comparative effectiveness of two different vaccines against COVID-19. Second, the findings are consistent with the indirect comparison of the effectiveness of these two vaccines based on the results of their respective clinical trials. Third, results from the sensitivity analyses using negative control outcomes confirmed that the effect of residual confounding, if present, is likely to be minimal, supporting the validity of our main findings. Fourth, considering that the time-varying confounders (e.g., COVID-19 vaccines where rollout in the period specified started in the oldest age groups) [37] may impact the results, we divided the participants’ enrolment period into weekly time blocks when they received either the BNT162b2 or ChAdOx1 nCoV-19 vaccines. In each time block we calculated propensity score (PS) and adopted an overlap weighting approach in each weekly time block to balance the baseline characteristics.

However, our study also has some limitations. First, although we used rigorous approaches to control for confounding (i.e., demographic characteristics, medical history, comorbidities, and health care utilization), unmeasured residual confounding, such as the severity of comorbidities, cannot be ruled out. Second, owing to a lack of detailed information in IMRD, we are unable to assess the comparative effectiveness of two vaccines on several other sequelae of COVID-19, such as intensive care unit admission, mechanical ventilation, or length of hospitalisation. Future studies are needed to evaluate these additional outcomes. Third, we cannot access the data held in the hospital and were not reported back to GPs (e.g., tests were performed at the hospital and were not reported back to GPs). Thus, misclassification of the COVID-19 diagnosis could occur and bias the study findings. However, such bias, if occurred, is likely to be small and non-differential. As a result, it would bias the observed associations towards the null. Moreover, although the original PCR test results were not available and the sensitivity for capturing COVID-19 cases through the Read Code has not been validated in the IMRD, a recent study of COVID-19 codes recorded in the primary care setting suggested that clinical diagnosis of COVID-19 by physicians followed a similar trend to test positive cases confirmed by the UK national testing service [38]. Fourth, we could only access data within the GP system. Thus, we were unable to evaluate the comparative effectiveness of BNT162b2 and ChAdOx1 nCoV-19 vaccines among individuals who were not registered with a GP or whose vaccination record was not captured by their GP. Future studies are needed to assess whether the effectiveness of BNT162b2 and ChAdOx1 nCoV-19 vaccine varies among this population.

### Clinical implications

This emulation of a target trial of a head-to-head comparison of two vaccines, provides evidence that the effectiveness of the BNT162b2 against COVID-19 appears better than the ChAdOx1 vaccine for both SARS-CoV-2 infection and hospitalisation for COVID-19 but not death from COVID-19. The percentage of fully vaccinated population remains low (<20%) in most countries of Africa and several countries of West Asia [6]. Although our findings suggest BNT162b2 vaccine may be preferred to reduce the SARS-CoV-2 infection rate and hospitalisation for COVID-19, the choice of vaccine type should not only be based on the potential differences in effectiveness. The decision regarding which COVID-19 vaccine to receive should consider other factors including availability, cost, and individual characteristics [39].

## Conclusions

In this population-based data, the effectiveness of the BNT162b2 against COVID-19 appears better than the ChAdOx1 vaccine for SARS-CoV-2 infection and hospitalisation for COVID-19. Although our findings suggested that the BNT162b2 vaccine may have a slightly better effect on lowering the SARS-CoV-2 infection rate and hospitalisation for COVID-19 than the ChAdOx1 nCoV-19 vaccine, both vaccines are effective against COVID-19 and its severe sequelae and should be encouraged to receive whichever vaccine is available.

## Supplementary Information

**Additional file 1: Table S1.** Read codes used for covid-19 vaccination records and diagnosed SARS-CoV-2 infection. **Figure S1.** A directed acyclic graph of the comparative effectiveness of BNT162b2 and ChAdOx1 nCoV-19 vaccines against COVID-19 and potential confounders.

## Data Availability

Available for purchase from info@the-health-improvement-network.co.uk.

## Abbreviations

AdV: Adenovirus
IMRD: IQVIA Medical Research Database
GPs: General practitioners
PS: Propensity score
BMI: Body mass index
RD: Rate difference
HR: Hazard ratio
CI: Confidence interval
