# Supplementary material for: Comparative effectiveness of BNT162b2 and ChAdOx1 nCoV-19 vaccines against COVID-19
Source: BMC Med. 2023 Feb 28;21:78. doi: 10.1186/s12916-023-02795-w (PMC9974059; doi:10.1186/s12916-023-02795-w)
Supplement: Supplementary file 1 — Additional file 1: Table S1. Read codes used for covid-19 vaccination records and diagnosed SARS-CoV-2 infection. Figure S1. A directed acyclic graph of the comparative effectiveness of BNT162b2 and ChAdOx1 nCoV-19 vaccines against COVID-19 and potential confounders. [file 12916_2023_2795_MOESM1_ESM.docx]

**Table S1. Read codes used for covid-19 vaccination records and diagnosed SARS-CoV-2 infection**

|  | **Codes** |
| --- | --- |
| **BNT162b2 Vaccination** | 1022010134, 1022010140 |
| **ChAdOx1 Vaccination** | 1022010136, 1022010139 |
| **Other Vaccination** | 1022010135, 1022010137, 1022010138, 1022010142, 1022010143 |
| **Diagnosed SARS-CoV-2 infection** | 43hF.00, 43kB100, 4J3R100, A076400, A795.00, A795100, A795200, A795300, A795400, A795500, A7y0000, AyuDC00, AyuJC00, AyuKL00, F289.00, F529.00, G520800, G558500, H051100, H204.00 |


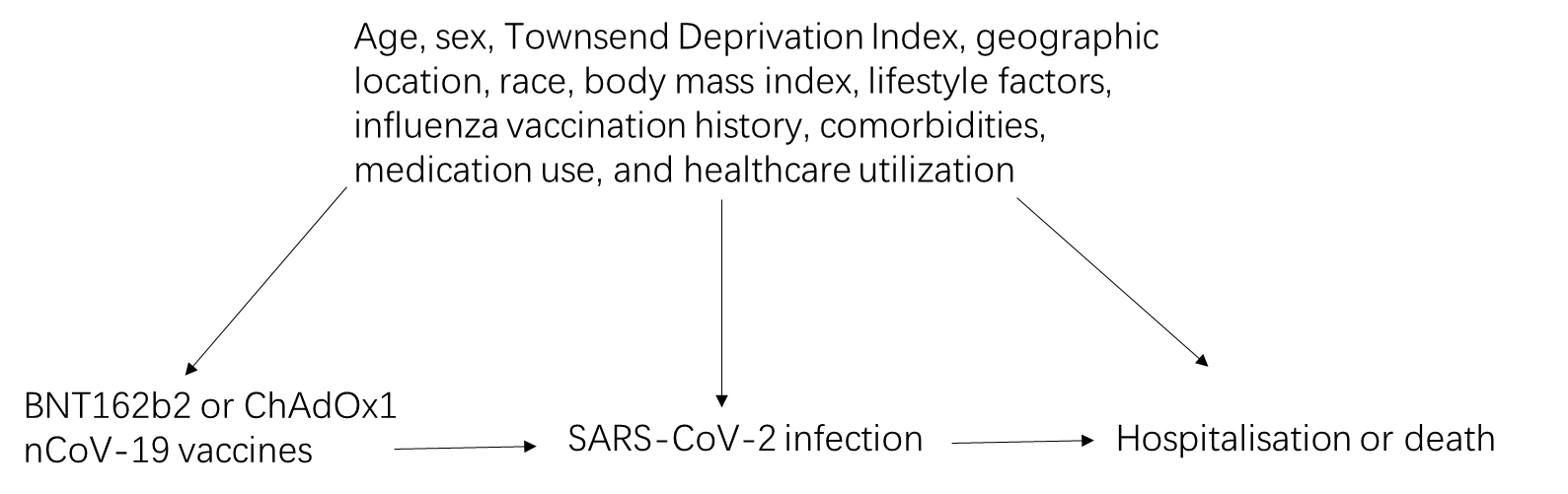


**Figure S1**. A directed acyclic graph of the comparative effectiveness of BNT162b2 and ChAdOx1 nCoV-19 vaccines against COVID-19 and potential confounders.
